# Genomic and Metagenomic Insights Into the Microbial Community in the Regenerating Intestine of the Sea Cucumber *Apostichopus japonicus*

**DOI:** 10.3389/fmicb.2019.01165

**Published:** 2019-06-04

**Authors:** Hongxia Zhang, Qing Wang, Shilin Liu, Da Huo, Jianmin Zhao, Libin Zhang, Ye Zhao, Lina Sun, Hongsheng Yang

**Affiliations:** ^1^CAS Key Laboratory of Marine Ecology and Environmental Sciences, Institute of Oceanology, Chinese Academy of Sciences (CAS), Qingdao, China; ^2^Laboratory for Marine Ecology and Environmental Science, Qingdao National Laboratory for Marine Science and Technology, Qingdao, China; ^3^Center for Ocean Mega-Science, Chinese Academy of Sciences (CAS), Qingdao, China; ^4^CAS Key Laboratory of Coastal Environmental Processes and Ecological Remediation, Yantai Institute of Coastal Zone Research, Chinese Academy of Sciences (CAS), Yantai, China; ^5^University of Chinese Academy of Sciences, Beijing, China; ^6^Ocean School, Yantai University, Yantai, China

**Keywords:** *Apostichopus japonicus*, intestine regeneration, intestinal microbiota, genomic, metagenomic

## Abstract

Host-intestine microbiota interactions have been widely studied in aquatic animals, but these interactions in the intestine regeneration process of the sea cucumber *Apostichopus japonicus* have been rarely investigated. To understand how intestine regeneration impacts the developing intestinal microbiome composition and function, we performed a case study to characterize the intestinal microbial composition and functional genes of *A. japonicus* during intestine regeneration stages. High-throughput 16S rRNA gene sequencing revealed significantly different intestine microbiota compositions in different regeneration stages. The phylogenetic diversity and composition of the intestinal microbiota changed significantly in the early regeneration stage and tended to recover in the end stage. During the regeneration process, the abundance of Bacteroidetes and Rhodobacterales increased significantly. A network analysis revealed that Rhodobacteraceae and Flavobacteriaceae may function as keystone taxa in the intestinal microbial community of *A. japonicus* during intestine regeneration. Metagenomic analyses of representative samples revealed that the microbiomes of regenerating intestines were enriched in genes facilitating cell proliferation, digestion and immunity. The increased abundance of Bacteroidetes elevated the enrichment of genes associated with carbohydrate utilization. Some functional features in the subsystem category changed in a pattern that was consistent with the changing pattern of microbiota composition during intestine regeneration. Our results revealed that seemingly regular alterations in the intestinal microbiome composition and function are associated with intestine regeneration stages. Intestinal microbiota can increase the abundance of beneficial bacterial members and upregulate related functional genes to adapt to intestine regeneration and reconstruct a stable community structure. This study provides a new insight into the mechanism of the host-microbiota interaction response to organ regeneration.

## Introduction

Intestinal microbiome, which exert important influences on a host’s development and tissue physiology, immune regulation, metabolic absorption and so on, have been widely studied in many organisms. For instance, intestinal bacteria can enhance the stability of β-catenin in intestinal epithelial cells and promote rates of cell proliferation in the vertebrate intestine (Cheesman et al., 2011). A previous study also found that different types of intestinal microbiota enhanced the healing of intestinal anastomoses in mice (Okada et al., 1999). In addition, recent research has revealed that the intestinal microbiome modulates animal behaviors, and shapes various aspects of basic neurodevelopmental processes, such as the formation of the blood-brain barrier, neurogenesis, myelination, and microglia maturation (Sharon et al., 2016).

The sea cucumber *Apostichopus japonicus* is an economically important fishery resource along the East Asian coast. Sea cucumbers possesses high nutritional and economic value as they are abundant in protein and mucopolysaccharides, and their market demand is high; therefore, the scale of farming is growing rapidly. *A. japonicus* ingests organic matter, microbes, algae, protozoa as well as aquatic animal detritus and thus affects the benthic material recycling (Choo, 2008; Yamazaki et al., 2016). The aforementioned broad-spectrum characteristics of intestinal microbiota define them as integral contributors to the development, growth and physiological health of the sea cucumber *A. japonicus*. In the past decade, studies have focused on (i) bacterial community structures in the intestine of *A. japonicus* (Gao et al., 2014a,b); (ii) the physiological characterization of intestinal bacterial isolates (Zhang et al., 2013; Wang et al., 2015); and (iii) the potential effects of intestinal bacteria on sea cucumber growth. For instance, studies report that larger and smaller *A. japonicus* individuals have significantly different intestinal microbial communities (Sha et al., 2016; Yamazaki et al., 2016). Recent studies have attempted to explain how intestinal microbiota are shaped by external factors, such as probiotics, diet and habitat types (Yang et al., 2015; Qi et al., 2017; Wang et al., 2017; Zhao et al., 2018; Ma et al., 2019). However, internal factors of organisms that may contribute to intestinal microbiota modulation have been scarcely explored.

Sea cucumbers are an excellent model to study organ regeneration since they possess a unique biological mechanism called evisceration when they are subjected to natural or induced stimulation (Dolmatov and Ginanova, 2009; Li et al., 2017b; Sun et al., 2017a). The lost organs, including the intestines, hemal system and respiratory trees, can concurrently regenerate within a few weeks (Shukalyuk and Dolmatov, 2001; Sun et al., 2011). Regeneration is a fascinating biological event that has aroused interest in many researchers. For the past two decades, studies have investigated many mechanisms of regenerative processes in sea cucumbers, including morphological features, cell division, dedifferentiation, cell proliferation and migration, nerve regrowth, and molecular regulatory mechanisms (García-Arrarás et al., 1998; Dolmatov and Ginanova, 2009; Sun et al., 2011, 2017a, 2018; Sun L.N. et al., 2013; Li et al., 2017b; Zhang et al., 2017). Moreover, the intestine regeneration process is accompanied by the reconstruction of the intestinal microbial community. To the best of our knowledge, only one study thus far has addressed the question of variation in the intestinal bacterial composition of *A. japonicus* during intestine regeneration stages (Wang et al., 2018). This study found that the bacterial community structure in intestine of *A. japonicus* varied greatly in different regeneration stages, with a significant difference between the earlier and later stages. Therefore, little is known about how such life events impact the development of the intestinal microbiome composition and function.

Previous studies have shown that hosts can develop tolerance to intestinal microbial antigens early in life and then maintain a mutually beneficial relationship with intestinal microbiota (Knoop et al., 2017). Adult intestinal microbiota are thought to be relatively stable over time, and this stability imparts resilience to various perturbations, ensuring the host’s continued intestinal function (Koenig et al., 2011). Therefore, we are interested in how the intestinal microbiota recover such stability and resilience. Understanding the regulatory mechanisms of microbial associations in the regenerating intestine may help develop a theoretical basis for further understanding the role of intestinal microbiome in the process of tissue and organ regeneration.

We analyzed the compositions and functional characteristics of microbial community in the regenerating intestine of *A. japonicus* using 16S rRNA gene and metagenomic sequencing. These results were used to address the following questions: How does the intestinal microbiome composition and function respond to intestinal regeneration? What are the keystone taxa in the intestinal microbial community of *A. japonicus* during intestine regeneration? This analysis allows us to pinpoint specific events (intestine regeneration) likely associated with significant changes in the intestinal microbiome, which is of great theoretical significance to the healthy aquaculture of sea cucumbers and to the understanding of host-gut microbiota interactions.

## Materials and Methods

### Experimental Animals and Sample Collection

Adult *A. japonicas* (of 80–100 g) were collected in October 2017 from the coast of Weihai, Shandong, China. The animals were acclimated in seawater at 15 ± 1°C for 2 weeks prior to treatment and were fed a formulated diet once a day. After acclimation, evisceration was induced by injecting approximately 2 mL 0.35 M KCl into the coelomic cavity (Sun L. et al., 2013; Sun et al., 2017b). Eviscerated animals were kept in well-aerated indoor seawater tanks. We began recording observations at the point when the sea cucumber had expelled the entire intestine. At least 9 individuals (three biological replicates × three individuals per biological replicate) per regeneration stage [10, 14, 18, and 21 days postevisceration (dpe), labeled as D10, D14, D18, and D21, respectively] were used for analyses. Sea cucumbers that did not undergo evisceration were used as the control (three biological replicates, labeled as CK). After 7 days of intestine regeneration, lumen formation of the new intestine began; the intestine gradually developed to form a complete structure in which the digestive and absorptive functions were restored during days 14–21 of intestine regeneration (Sun et al., 2011; Sun L. et al., 2013). The 10th day was therefore an appropriate time point for sampling the new intestine.

For intestine collection, 9 *A. japonicus* individuals were randomly sampled in different regeneration stages. Individuals were rinsed with sterile seawater and moved into sterile plates. The coelomic fluid was withdrawn from the coelom of sea cucumbers using sterile syringes. The intestines were then aseptically dissected and transferred into sterile tubes, and preserved at −80°C until DNA extraction.

**Table 1:** Summary of the alpha diversity of bacterial communities in samples from different regeneration stages.

Sample ID	OTU	Chao 1	Coverage	Shannon	Simpson
CK	766	1275 (1143, 1454)	0.992879	2.95 (2.93, 2.97)	0.1713 (0.1686, 0.1741)
D10	142	195 (163, 272)	0.998928	2.22 (2.2, 2.24)	0.2873 (0.2819, 0.2927)
D14	920	1181 (1109, 1280)	0.992959	4.47 (4.45, 4.49)	0.0316 (0.0309, 0.0322)
D18	916	1205 (1129, 1309)	0.994295	4.09 (4.07, 4.11)	0.0519 (0.051, 0.0528)
D21	914	1312 (1208, 1452)	0.991934	4.06 (4.04, 4.09)	0.0817 (0.0796, 0.0839)

### DNA Extraction and 16S rRNA Gene Sequencing

Total DNA was extracted from the gut contents of *A. japonicas* by using a FastDNA SPIN Kit for Feces (MP Biomedicals, Santa Ana, CA, United States) in accordance with the instructions provided by the manufacturer. The extracted DNA was dissolved in 50 μL of TE buffer, quantified using a NanoDrop spectrophotometer (NanoDrop, Thermo Fisher Scientific, United States) and stored at −20°C prior to analysis. PCR amplification of bacterial 16S rRNA hypervariable regions V4–V5 was conducted using the universal primer set 515f (GTGCCAGCMGCCGCGGTAA) and 907r (CCGTCAATTCMTTTRAGTTT), with 5 bp barcodes fused to the forward primer to allow sample multiplexing. The purified PCR products with different barcodes were normalized in equimolar amounts and then prepared using an NEB Next^®^Ultra^TM^DNA Library Prep Kit for Illumina (NEB, United States) following the manufacturer’s protocol and sequenced on an Illumina HiSeq platform.

### Deep Sequencing Data Processing

Raw deep sequencing data were processed using Quantitative Insights Into Microbial Ecology software (QIIME version 1.9.0^1^; Caporaso et al., 2010) with the default parameters unless otherwise noted. After all chimeric and low-quality reads were removed, qualified sequences were clustered into operational taxonomic units (OTUs) at the 97% identity threshold level, and the most abundant sequence from each OTU was chosen as a representative sequence for that OTU. Taxonomic classification of each OTU was assigned using the Ribosomal Database Project classifier. The average relative abundance (%) of predominant genus-level taxonomic groups in each sample was estimated by comparing the number of sequences assigned to a specific taxon vs. the number of total sequences obtained for that sample. Based on the OTU numbers, principal components analysis (PCA) was performed using R version 3.0.2 (package *stats*) to test the differences in microbial community structures.

### Metagenomic Sequencing

Each representative DNA sample from the sea cucumber intestine for which sufficient volumes were available after 16S typing was used for metagenomic sequencing. Metagenomic DNA paired-end libraries were prepared with an insert size of 350 bp and were quantified using a Qubit Fluorometer (Life Technologies, Carlsbad, CA, United States) and Agilent 2100 bioanalyzer (Agilent Technologies, Palo Alto, CA, United States). Sequencing was performed on an Illumina HiSeq PE150 platform.

Raw reads were preprocessed using FasqMcf to exclude adapter sequences and low-quality sequences (Aronesty, 2011), and reads derived from host contamination were filtered using bowtie 2.2.4 (Langmead and Salzberg, 2012) with the parameters “-end-to-end, -sensitive, -I 200, -X 400.” Clean reads were assembled and analyzed by SOAPdenovo software (V2.04) (Luo et al., 2012) using the following parameters “-d 1, -M 3, -R, -u, -F, -K 55.” Then, gene prediction was performed on contigs larger than 500 bp by MetaGeneMark software with the default parameter, and gene models with CDS lengths less than 100 bp were filtered out (Qin et al., 2010; Nielsen et al., 2014). The gene catalog was constructed using the gene models predicted from each sample by CD-HIT-EST (version 4.6.6) (Li and Godzik, 2006) with the parameter “-c 0.95 -n 10 -G 0 -aS 0.9,” which adopts a greedy incremental clustering algorithm and criteria as identity >95% and overlap >90% of the shorter genes.

### Metagenomic Analysis

DIAMOND software (version 0.9.9) (Buchfink et al., 2015) was used to align the unigenes to the sequences of bacteria, fungi, archaea and viruses, which were all extracted from the NCBI nr database (Version: 2018-01-02) with the parameter “-e 1e-5.” The functional assignments of protein sequences were made on the basis of DIAMOND alignment against the KEGG protein database (version 2018-01-01) (Minoru et al., 2006), eggNOG database (version 4.5) (Sean et al., 2014), and CAZy database (Version 20150704) (Cantarel et al., 2009) by using the best hit with an *e* < 1e-5. For each sequence’s BLAST result, the best BLAST hit was used for subsequent analysis.

Phylum, class, order, family, genus, species, KEGG orthology (KO), and orthologous group (OG) relative abundances were calculated by summing the abundance of the respective genes belonging to each category per sample based on the taxonomic assignments, KO and OG annotations, respectively. The relative gene abundance profile was also summarized into KEGG, eggNOG and CAZy functional profiles for the functional analysis.

### Accession Numbers of Nucleotide Sequences

All 16S rRNA gene and metagenomic sequence raw data were submitted to the sequence read archive (SRA) with project accession numbers PRJNA512056 and PRJNA518164.

## Results

### 16S rRNA Gene Analysis Revealed a Temporal Pattern of Bacterial Diversity

At 97% sequence identity, a total of 1,671 OTUs were obtained across all samples. The samples from the CK group contained 766 OTUs (on average), while D10, D14, D18, and D21 regeneration samples contained 142, 920, 916, 914 OTUs (on average), respectively (Table 1). We analyzed both shared and unique OTUs in the bacterial communities of *A. japonicus* intestines during regeneration stages to further identify the dominant microbiota. Samples from different regeneration stages shared only 14 OTUs (Supplementary Figure 1 and Supplementary Table 1). Lower values for community richness were observed in the early regeneration stage (D10).

The alpha diversity of the bacterial community varied greatly in different regeneration stages. Shannon indices varied from 2.22 to 4.47, and Chao1 richness ranged from 195 to 1,312 OTUs in all samples (Table 1). The earliest stage (D10) had the lowest Shannon index and Chao1 richness, with 2.22 and 195 OTUs, respectively; however, the diversity and richness gradually increased in later stages. These results indicated that there were significant differences in alpha diversity of bacterial community between different regeneration stages.

### Characterization of the Microbiome Composition in the Intestine of *Apostichopus japonicus* During Different Regeneration Stages

**FIGURE 1:**
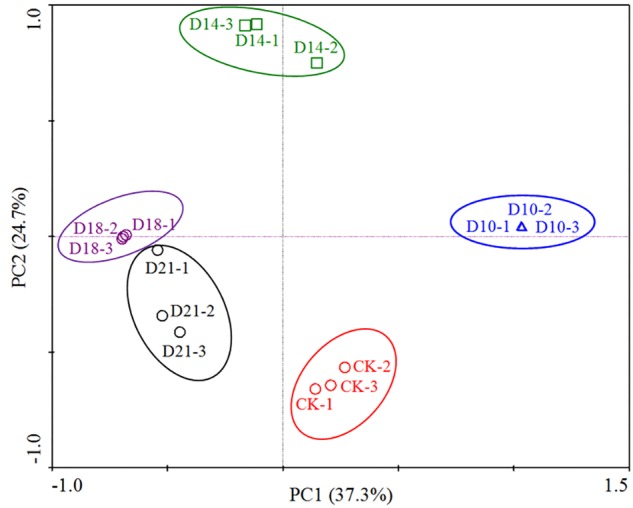
Principal component analysis (PCA) based on the relative abundance of 16S rRNA genes from 15 samples (5 sites × 3 replicates per stage). The scatterplot shows principal coordinate 1 (PC1) vs. principal coordinate 2 (PC2). The percentages of variation in the samples described by the plotted PCs are shown on the axes. The 3 replicates for each site are represented by a single color.

The bacterial communities in the intestine of sea cucumbers were classified as CK or regeneration stages based on a principal component analysis (PCA) of OTUs, which indicated that there were differences in bacterial community compositions. Figure 1 shows that 3 replicates from the same stage grouped together, and the bacterial community structures in different stages exhibited temporal patterns distinct from one other. Samples from the later stage (D21) close to the CK and other samples from different stages showed distinct separation, suggesting that the composition patterns of bacterial communities are associated with intestine regeneration stages.

**FIGURE 2:**
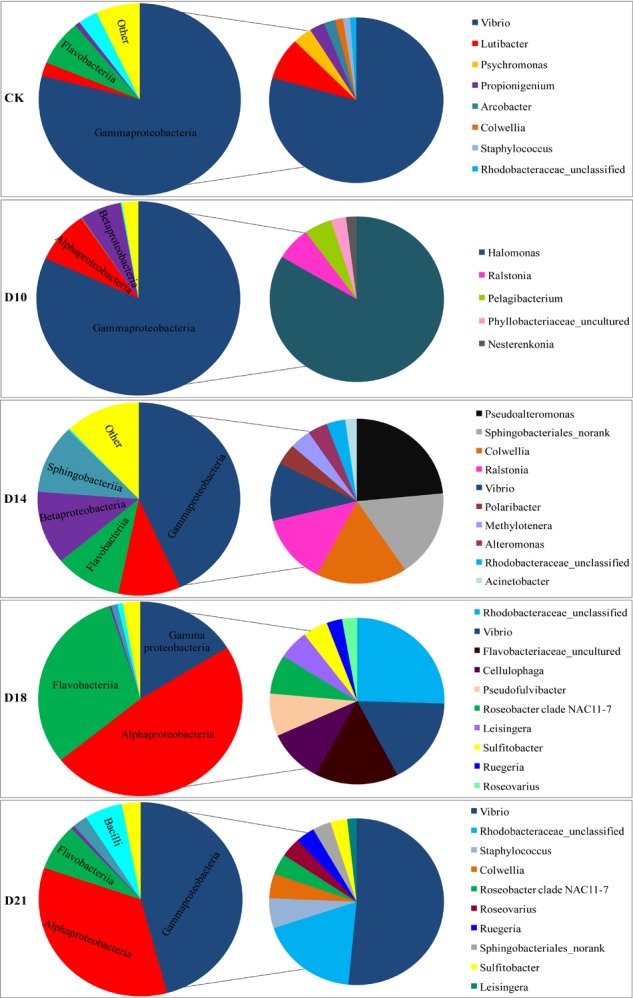
Composition of bacterial communities in the intestine of the sea cucumber *Apostichopus japonicus* during different regeneration stages (%). Note: The big pie chart is the class level, the small pie chart is the genus level.

Analysis of bacterial communities revealed distinct taxonomic compositions between intestine samples obtained from different regeneration stages (CK, D10, D14, D18, and D21) (Figure 2). In CK samples, Proteobacteria, Bacteroidetes and Firmicutes phyla predominated (96.2% of total OTU), as was also the case in D21 samples (Supplementary Figure 2). Most of the Proteobacteria reads were assigned as Gammaproteobacteria (average 78.9%), of which *Vibrio* was the dominant genus (71.5%). Flavobacteriia (7.7%), Bacilli (3.1%) and Alphaproteobacteria (2.3%) were the next most abundant classes. Flavobacteriia-related reads were predominantly affiliated with the genus *Lutibacter* (7.4%).

In the D10 samples, the Proteobacteria phylum (average 97.1%) was represented mostly by the Gammaproteobacteria (81.8%), Alphaproteobacteria (8.7%), and Betaproteobacteria (6.6%) classes, mainly related to *Halomonas* (10.7%), *Ralstonia* (6.4%), and *Pelagibacterium* (5.1%) genera. In the D14 samples, the Proteobacteria phylum (average 66.4%) was represented mostly by the Gammaproteobacteria (43.3%), Betaproteobacteria (12.2%), and Alphaproteobacteria (10.0%) classes, with *Pseudoalteromonas*, *Colwellia*, *Ralstonia*, and *Vibrio* comprising 14.2, 10.3, 8.2, and 6.9% of the total, respectively. Bacteroidetes (average 22.3%) was the next most abundant phylum, represented mainly by the Sphingobacteriia (11.5%) and Flavobacteriia (10.7%) classes and mainly related to the Sphingobacteriales_norank (10.4%) genus.

The composition of the intestinal bacterial community varied greatly in the D18 samples at the class and genus levels. Within the Proteobacteria (64.6%, on average) phylum, the Alphaproteobacteria (48.0%) and Gammaproteobacteria (16.2%) classes predominated. Of these, 18.6% of the reads were assigned as unclassified Rhodobacteraceae, 12.4% as *Vibrio*, and 5.5% as *Roseobacter* clade NAC11-7. Bacteroidetes (average 31.9%) were represented mainly by the Flavobacteriia (30.89%) class and were mainly related to the Flavobacteriaceae_uncultured (11.3%), *Cellulophaga* (7.5%), and *Pseudofulvibacter* (6.0%) genera.

Compared to CK samples, a marked recovery profile was detected in D21 samples (Figure 2 and Supplementary Figure 2). Within the Proteobacteria, which corresponded to 84.9% of all the reads in D21 sample, the Gammaproteobacteria (45.8%) and Alphaproteobacteria (34.5%) classes prevailed, with *Vibrio*, unclassified Rhodobacteraceae and *Colwellia* comprising 35.0, 12.5, and 3.2% of the total, respectively. Firmicutes (62.3%) were represented mainly by the Bacilli (6.0%) class, and *Staphylococcus* (4.0%) genus. Bacteroidetes was also present (10.2%), mainly related to the Flavobacteriia (7.8%) class, and the Sphingobacteriales_norank (2.3%) genus.

### Potential Key Players in the Regenerating Intestinal Microbiome

**FIGURE 3:**
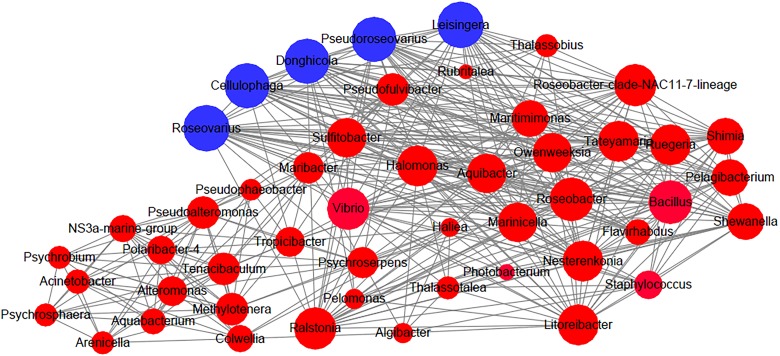
Network analysis for connection among bacterial groups in the intestine of the sea cucumber *Apostichopus japonicus* during the regeneration process. Note: Blue nodes: the high mean degree taxa. The size of the nodes is proportional to the connected degree.

Recently, the microbial network has become an increasingly popular tool to analyze microbial community structure. Analyses of microbial networks can help researchers identify high mean degree taxa and further predict keystone species and species interactions. Here, we constructed a co-occurrence network by using all samples during the intestine regeneration process and attempted to identify potential key players during the regeneration period in this study. *Roseovarius*, *Leisingera*, *Cellulophaga*, *Pseudoroseovarius*, and *Donghicola* were found to be high mean degree taxa (Figure 3) and may be key players in the intestinal microbial community of *A. japonicus* during the regeneration stages.

### Metagenomic Analysis Revealed Functional Differentiation Among the Different Regeneration Stages

Metagenome sequencing using an Illumina HiSeq platform was performed on intestinal specimens during different regeneration stages. To detect the microbial function response to intestine evisceration and regeneration, 4 time points (the beginning, middle and end of intestinal regeneration, and the CK) were chosen. From the CK and D10, D14, D21 samples, 89,430,618 reads (13.4 Gb), 87,521,564 reads (13.1 Gb), 92,642,774 reads (13.8 Gb), and 88,435,042 reads (13.2 Gb), respectively, were obtained. After quality filtering and removing host sequences, 11,089 reads, 11,559 reads, 11,644 reads and 11,558 reads from CK and the D10, D14, D21 stages, respectively, were used for MG-RAST annotation. Bacterial reads occupied 48.3–50.2%, eukaryotic reads occupied 45.4–47.7%, virus reads occupied 2.4–6.2%, and archaeal reads occupied 0.17–0.18% of all libraries.

**FIGURE 4:**
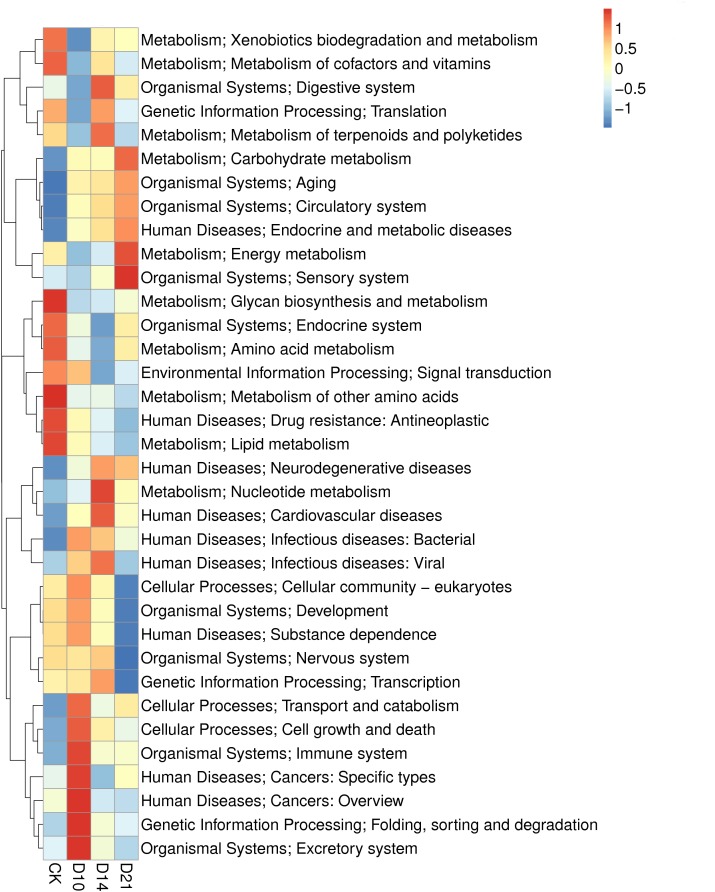
Heatmap of the KEGG analysis of intestinal microbiota in samples from different regeneration stages of the sea cucumber.

The metagenomic genes of samples from different regeneration stages (CK, D10, D14, and D21) were annotated with KEGG orthologous groups. The abundances of functional genes at different levels were calculated, and 238 functional genes were annotated to the metabolic pathway. The pathways of metabolism and human diseases contained the most abundant annotated genes, followed by the pathway of organism system (Supplementary Figure 4). The analysis showed that 23 KEGG functional features in the level 2 were more abundant in one of the samples. Genes annotated to “metabolism of other amino acids,” “lipid metabolism,” “glycan biosynthesis and metabolism,” “metabolism of cofactors and vitamins,” “drug resistance: antineoplastic” and others were significantly more abundant in the CK sample; and “excretory system,” “folding, sorting, and degradation,” “cancers: overview,” “cancers: specific types,” “immune system,” “cell growth and death,” and “transport and catabolism” were more abundant in the D10 sample. Regarding D14, genes annotated in “nucleotide metabolism,” “digestive system,” “metabolism of terpenoids and polyketides” and others were more abundant. For D21, the more abundant genes were annotated in “sensory system,” “energy metabolism,” and “carbohydrate metabolism” (Figure 4).

**FIGURE 5:**
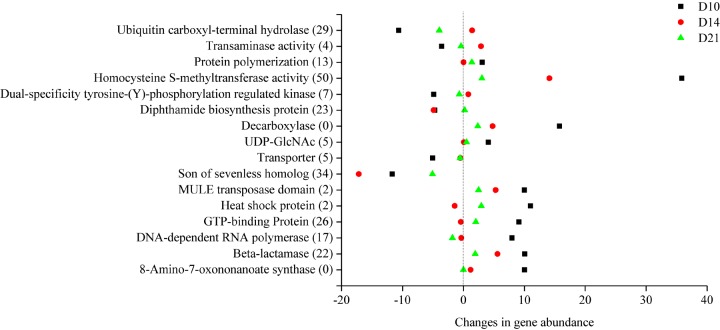
Changes in the functional gene abundance of intestinal microbiota in different regeneration stages.

In this study, metagenomic data were also compared with the eggNOG database to explore how the microbial gene functions varied in the intestine of *A. japonicus* during intestine regeneration. The functional features of the subsystem category at different levels were analyzed. The genes annotated to “amino acid transport and metabolism” and “replication, recombination and repair” were most abundant, followed by genes annotated to “posttranslational modification, protein turnover, chaperones,” “cytoskeleton,” and “signal transduction mechanisms” (Supplementary Figure 5). The gene abundance of eggNOG functions also changed during the different regeneration stages. As shown in Figure 5, 16 notable eggNOG functions occurred with a regular pattern during different regeneration stages. In detail, the abundance of genes responsible for homocysteine S-methyltransferase activity, son of sevenless homolog, ubiquitin carboxyl-terminal hydrolase, beta-lactamase, DNA-dependent RNA polymerase, homocysteine S-methyltransferase activity, heat shock protein, etc. in the level 2 subsystem category were up- or downregulated during the regeneration stages. These gene abundances varied significantly during regeneration stages; however, they tended to revert to the level of the CK specimens in the end stage.

## Discussion

Intestinal microbiota have been recognized as important players in sea cucumber health and have broad effects on growth and function (Yamazaki et al., 2016; Li et al., 2017a). In recent years, questions concerning how external disturbances affect intestinal microbiota have been addressed, prompted by the development of 16S rRNA gene sequencing, powerful metagenomic analyses, and metabolomics technologies. The sea cucumber *A. japonicus* is an excellent model animal to study in terms of organ regeneration in host-microbiota interactions, however, little is known regarding how the intestinal microbiome composition and function vary in response to intestine regeneration. To the best of our knowledge, the present work is the first analysis of changes in intestinal microbial function during intestine regeneration stages in *A. japonicus*. We demonstrated that intestinal bacterial communities and microbial functional genes in *A. japonicus* varied significantly with a regular pattern during intestine regeneration stages. Because microbial quantity in the regenerating intestine of *A. japonicus* was decreased, fewer microbial reads were obtained by metagenomics in this study. However, metagenomic analyses can provide a better understanding of the microbiome function response to intestine regeneration events.

In this study, high-throughput sequencing results suggested that bacterial community structures in the intestine of *A. japonicus* showed significant differences in different regeneration stages. This result is consistent with a previous study (Wang et al., 2018). In the early stage (10 days), a sharp decline in the species richness and diversity of intestinal bacteria was observed, suggesting that the absence of an intestine led to the development of extensive and varied bacterial diversity in the intestine of *A. japonicus*. However, the diversity of intestinal bacteria then increased gradually. The intestinal bacterial community structures also changed at the phylum and class levels, and particularly at the genus level in the early stage, and then varied significantly in the later stages (14–18 days). Notably, the community composition recovered to a certain extent at the end of regeneration and was similar to the control (Figure 2 and Supplementary Figure 2), which is consistent with a previous report that bacterial community structure in the intestine of *A. japonicus* tended to be stable after 21 days of regeneration (Wang et al., 2018).

Proteobacteria was the main phylum observed in the intestine of *A. japonicus* (Gao et al., 2014a; Sha et al., 2016), and Bacteroidetes was the second most abundant phylum (Yang et al., 2015; Yamazaki et al., 2016; Wang et al., 2018) despite the variation in bacterial communities at lower taxonomic levels. In accordance with these previous studies, Proteobacteria was also the most abundant phylum in all samples in this work, particularly in the Gamma- (CK, D10, D14, and D21 samples) and Alphaproteobacteria (D18 sample) classes. *Vibrio* was the most dominant genus in the CK samples; more than 71.5% of sequences were assigned to *Vibrio*. Interestingly, *Vibrio* was absent in the D10 samples, and its relative abundance gradually increased to 35% (on average) and returned to the most dominant genus in the D21 samples. *Vibrio* is widely distributed in marine environments and the digestive tracts of aquatic animals, such as penaeid shrimps (Gomez-Gil et al., 1998) and zebrafish (Brugman et al., 2014). *Vibrio* was also reported as the predominant genus in the gut of *A. japonicus* in previous studies (Wang et al., 2018; Zhao et al., 2018). Several dozen *Vibrio* species have been shown to engage in a diversity of beneficial or pathogenic interactions with animal tissue (Ruby et al., 2005), and some species have been used as probiotics in aquaculture (Gao et al., 2014a). A previous study found that zebrafish T lymphocytes can effectively affect the intestinal microbial community structures by suppressing the outgrowth of *Vibrio* (Brugman et al., 2014). Similarly, the regenerating intestine of *A. japonicus* may present immunoselectivity, and thus the growth of *Vibrio* may have been suppressed in this study.

Importantly, the relative abundance of Bacteroidetes was lower in the early regeneration stage but gradually increased to the second most abundant phylum in the D14 and D18 samples and returned to similar levels in the end stage as those observed in the CK samples. In particular, Flavobacteriaceae_uncultured and Sphingobacteriales_norank prevailed in the D14 and D18 samples, respectively. Previous studies have reported that Bacteroides are specialized in acquiring and digesting a wide variety of polysaccharides (e.g., glycosyl hydrolases, cell-surface carbohydrate-binding proteins, fucoidan, etc.) (Sakai et al., 2002; Xu et al., 2003; Koenig et al., 2011). Flavobacteriaceae are extremely resistant to many antimicrobial agents, and they present low-grade pathogenicity (Jooste and Hugo, 1999). Flavobacteriaceae can produce carotenoids that have antioxidative activities (Shindo et al., 2007) and can utilize fucoidan, fucose, mannose, galactose and glucuronic acid (Sakai et al., 2002). Members of Sphingobacteriales that retain glycoside hydrolase genes can assimilate carbon from glucose and cellulose (Pinnell et al., 2014). The metabolic activities of Bacteroidetes might either directly or indirectly increase the production of carbohydrates, which is beneficial to the host during the intestine regeneration process. Thus, these results further support the notion that intestinal microbiota have regulatory mechanisms to adapt to the regenerating intestine that has an unsound function.

Discovering the keystone taxa, that is, members of the intestinal microbiota that may be important for microbial community structures and integrity, has been a recent focus of researchers interested in understanding intestinal microbial communities in many organisms (Banerjee et al., 2018; Röttjers and Faust, 2018). In the present study, we found that *Roseovarius*, *Leisingera*, *Cellulophaga*, *Pseudoroseovarius*, and *Donghicola* were the highly connected taxa in the intestinal microbial community of *A. japonicus* during the regeneration stages. Interestingly, *Roseovarius*, *Leisingera*, *Pseudoroseovarius*, and *Donghicola* belong to Rhodobacteraceae (within Rhodobacterales), and *Cellulophaga* belongs to Flavobacteriaceae (within Flavobacteriales). Rhodobacteraceae have been identified as keystone taxa in the microbial community of *Nannochloropsis salina* in aquatic ecosystems (Geng et al., 2016). Rhodobacteraceae species have also been frequently detected in the intestines of *A. japonicus* (Gao et al., 2014a; Sha et al., 2016; Wang et al., 2018). Moreover, dietary β-glucan supplementation can probably modulate the balance of intestinal microbiota and thus promote the proliferation of Rhodobacteraceae in the gut of sea cucumbers and activate the NF-kB signaling pathway (Yang et al., 2015). Yamazaki et al. (2016) found that Rhodobacterales retaining polyhydroxybutyrate (PHB) metabolism genes promoted the growth of *A. japonicus*. In this study, the abundance of Rhodobacterales was increased significantly in samples from D14 to D21 (Supplementary Figure 3). Additionally, 3 OTUs shared by all samples during intestine regeneration were affiliated to Rhodobacterales (Supplementary Table 1); thus, these OTUs might be important for the regrowth of the intestine. We predict that Rhodobacterales and Flavobacteriaceae may function as keystone taxa on the basis of their role in intestine ecosystems and their impact on microbial community structures.

Additionally, *Ralstonia* (within Burkholderiales), *Pelagibacterium*, and *Aliihoeflea* (within Rhizobiales) were also present in all samples during the whole regeneration process. A previous review reported that many members of Rhizobiales and Burkholderiales were consistently identified as the keystone taxa in different ecosystems (Banerjee et al., 2018). They may play an important role in the regenerating intestine of the sea cucumber, and future studies are needed to evaluate this putative role in microbial functions.

Metagenomic data provided additional insight into the dynamics of the regenerating microbiome function. For example, the abundance of Rhodobacterales was increased significantly in the later regeneration stages. At that time, genes annotated to *pha*A (acetyl-CoA C-acetyltransferase), which is essential in PHB synthesis from acetyl-CoA to PHB (Yamazaki et al., 2016), were correspondingly enriched (data not shown). These results suggest that a transient variation in the intestinal microbiota might have been directly related to the regeneration activity. Another noteworthy observation was that functional features in the subsystem category were changed in a regular pattern. The abundance of functional genes was up- or downregulated clearly during the regeneration process (Figure 5); however, the expression tended to revert to the level of the CK samples in the end stage of regeneration. This pattern was observed in a previous study (Sun L.N. et al., 2013), which reported that the expression level of *Wnt*6 in the regenerating intestine of the sea cucumber *A. japonicus* was upregulated at 7 and 14 days and then gradually decreased to the control level at 21 days. These results together suggest that gene expression in the intestine and intestinal microbiota showed the same changing trend in response to intestine regeneration in *A. japonicus*. In addition, during the regeneration process, functional genes responsible for cell growth and death, carbohydrate metabolism, the digestive system and the immune system were upregulated (Supplementary Figure 6), which is in accord with shifts in specific microbial communities. Together, these results suggest that intestinal microbiota can regulate the abundance of beneficial members and related functional genes, which are helpful for microbial community reconstruction and intestine regrowth in *A. japonicus* during regeneration. In addition, enriched eukaryotic functional genes reflected high relative levels of fungi in the present study; hereafter, additional experiments should be conducted to further assess how intestinal fungi respond to special life events.

## Conclusion

In conclusion, this study revealed that life events can alter the taxonomic and functional features of the intestinal microbiome. We observed large alterations in the abundance of major groups and functional genes; interestingly, these alterations were associated with the intestine regeneration stages. The diversity of the intestinal microbiota was decreased significantly in the early regeneration stage and then increased gradually and recovered in the end stage. The bacterial community composition changed significantly in the regeneration process and tended to recover in the end stage. Network analysis revealed that Rhodobacterales and Flavobacteriaceae may function as key players in maintaining the stability of the community structure during the intestine regeneration stages. Metagenomic analysis revealed that the increased abundance of Bacteroidetes elevated the enrichment of genes associated with carbohydrate utilization. The intestinal microbiome was enriched in genes facilitating cell proliferation, digestion, and immunity during the regeneration stages. Some gene expression in the intestine and intestinal microbiota showed the same changing trend in response to intestine regeneration in *A. japonicus*. Our study indicated that intestinal microbiota have regulatory mechanisms to adapt to the regenerating intestine and provides insights into the host-microbiota interaction response to organ regeneration. The more exact regulatory mechanisms of intestinal microbial composition and functions and their effect on the host remain to be analyzed.

## Data Availability

The datasets generated for this study can be found in sequence read archive, PRJNA512056 and PRJNA518164.

## Author Contributions

HZ, QW, SL, JZ, LZ, LS, and HY contributed to the presented idea and design. HZ implemented the computational and statistical analyses and took the lead in writing the manuscript. DH assisted with data analysis. LS and HY supervised the findings of this work. All authors provided critical feedback and helped to conduct the research, analysis and manuscript.

## Conflict of Interest Statement

The authors declare that the research was conducted in the absence of any commercial or financial relationships that could be construed as a potential conflict of interest.

## Footnotes

1 http://www.qiime.org
