# Supplementary material for: Genomic and Metagenomic Insights Into the Microbial Community in the Regenerating Intestine of the Sea Cucumber Apostichopus japonicus
Source: Front Microbiol. 2019 Jun 4;10:1165. doi: 10.3389/fmicb.2019.01165 (PMC6558059; doi:10.3389/fmicb.2019.01165)
Supplement: Supplementary file 1 [file Data_Sheet_1.docx]

**Supplementary Material**

**SUPPLEMENTARY TABLES AND FIGURES**

**Supplementary Table 1** | Summary of the 14 shared OTUs and their read numbers in all samples

| OTU ID | Order | Genus | CK | D10 | D14 | D18 | D21 |
| --- | --- | --- | --- | --- | --- | --- | --- |
| OTU5 | Oceanospirillales | *Halomonas* | 10 | 15313 | 79 | 7 | 3 |
| OTU6 | Burkholderiales | *Ralstonia* | 143 | 1035 | 1695 | 18 | 16 |
| OTU18 | Rhizobiales | *Pelagibacterium* | 18 | 1443 | 121 | 2 | 1 |
| **OTU19** | **Rhodobacterales** | **Rhodobacteraceae_Unclassified** | **28** | **1** | **160** | **336** | **455** |
| OTU21 | Burkholderiales | *Ralstonia* | 13 | 310 | 180 | 2 | 3 |
| OTU27 | Burkholderiales | *Ralstonia* | 23 | 217 | 253 | 3 | 2 |
| OTU31 | Burkholderiales | *Ralstonia* | 17 | 219 | 216 | 3 | 1 |
| **OTU47** | **Rhodobacterales** | **Rhodobacteraceae_Unclassified** | **118** | **34** | **155** | **482** | **397** |
| **OTU58** | **Rhodobacterales** | ***Litoreibacter*** | **5** | **33** | **130** | **211** | **456** |
| OTU66 | Burkholderiales | *Pelomonas* | 9 | 35 | 151 | 2 | 1 |
| OTU78 | Rhizobiales | Phyllobacteriaceae_uncultured | 2 | 785 | 18 | 1 | 2 |
| OTU120 | Rhizobiales | *Aliihoeflea* | 2 | 42 | 13 | 4 | 7 |
| OTU260 | Lactobacillales | *Enterococcus* | 14 | 1 | 1 | 3 | 17 |
| OTU304 | Oceanospirillales | *Halomonas* | 4 | 31 | 7 | 20 | 5 |

**Supplementary Figure 1** | A Venn diagram showing the numbers of bacterial OTUs in samples from different regeneration stages.

**Supplementary Figure 2** | Relative abundance of bacterial communities at the phylum level in the intestine of the sea cucumber *Apostichopus japonicus* during the different regeneration stages.

**Supplementary Figure 3** | Relative abundance of bacterial communities at the order level in the intestine of the sea cucumber *Apostichopus japonicus* during the different regeneration stages.

**Supplementary Figure 4** | KEGG analysis of intestinal microbiota during the different regeneration stages.

**Supplementary Figure 5** | Comparison of eggNOG functional profiles of the intestinal microbiota during the different regeneration stages.

**Supplementary Figure 6** | Comparison of the KEGG functional profiles of the intestinal microbiota during the different regeneration stages.
